# Biofilm-Forming Ability of Infectious Organisms on Biomimetic Surfaces—An *In Vitro* and Machine-Learning Analysis

**DOI:** 10.1021/acsomega.5c04335

**Published:** 2025-08-25

**Authors:** Geetha Venkatachalam, Nandakumar Venkatesan, Shloak Vatsal, Indira Chavan, Arnab Bakshi, Mukesh Doble

**Affiliations:** † Ecogreen Innovations Pvt Ltd, Nirmaan, The Pre-Incubator, Sudha Shankar Innovation Hub, IIT Madras, Chennai 600036, India; ‡ Sri Ramachandra Faculty of Engineering and Technology, 204733 Sri Ramachandra Institute of Higher Education and Research, Chennai 600116, India; § Theevanam Additives and Nutraceuts Pvt. Ltd, IITM Research Park, Chennai 600113, India; ∥ Department of Cariology, Saveetha Dental College, SIMATS, Chennai 600077, India

## Abstract

The current study explores the adhesion and biofilm-forming ability of different opportunistic pathogens including *Staphylococcus aureus*, *Escherichia coli*, *Lactobacillus* spp., *Streptococcus mutans*, and *Pseudomonas aeruginosa* on lotus leaf (LL) and peepal leaf (PL) inspired biomimetic hydrophobic surfaces. Surface topology that mimics the respective leaves was fabricated using polylactic acid by solvent casting. Water contact-angle measurements revealed varying degrees of material surface hydrophobicity with respect to the varying surface roughness. The biofilm formation was significantly influenced by the type of polymer surface (*p* < 0.005) and the hydrophobicity of the bacterial surface (*p* < 0.0001). Multilayer perceptron (MLP), a feed-forward neural network, gave the best results with 5-fold cross-validation and an accuracy of 85%. J48-base model predicted that organisms with a surface hydrophobicity of >57% had higher biofilm-forming ability than others. Similarly, polymers with low surface roughness (roughness < 0.46) had reduced biofilm formation. In conclusion, biomimetic hydrophobic surfaces reduce the biofilm formation on implants.

## Graphical abstract

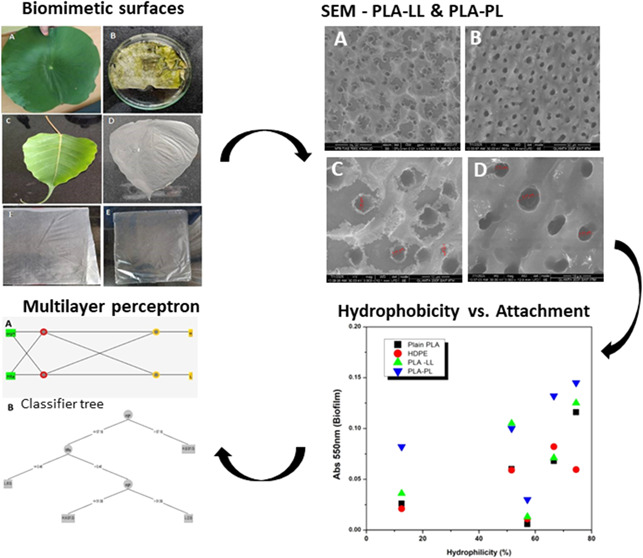

## 1 Introduction

*Nelumbo nucifera* (lotus) leaves are superhydrophobic (contact angle > 140°) semiaquatic plants with peltate leaves of ∼30 cm diameter and possess excellent water repellency. The superhydrophobicity could be attributed to the hierarchical structure in the upper epidermis made up of papillae densely coated with agglomerated wax tubulus^1^ that prevent wetting. The papillae and tip radii of other plant leaves have a larger diameter with waxy platelets or a waxy film.^2^ *Ficus religiosa* (peepal) leaves are hydrophilic in nature with a contact angle of <90°, have a smooth surface, and lack micro- and nanostructures.^3^ Bacterial biofilm formation on tissue surfaces and implants is critical in the clinical setting because these biofilms develop resistance to antimicrobial agents and cause persistent infections. Designing bioinspired materials that can repel water adhesion and reduce bacterial adhesion without inducing any antimicrobial resistance is the need of the hour. Several works have reported that mimicking bioinspired surfaces, including rose petals,^4^ rough shark skin pattern,^5^ biomimetic PMMA coating,^6^ and fabrication of PDMS surfaces^7^ with micropatterns, which reflects antibiofilm effect, led to the development of several biomaterials that are bactericidal without inducing antimicrobial resistance.

Biofilms are bacterial cells embedded in a self-produced extracellular matrix (EPS) containing 5–25% bacterial cells and 75–95% glycocalyx matrix, facilitating infection and bacterial survival in diverse environments. The oral microflora includes 700 bacterial species that settle on the hard palate, oral mucosa, teeth, tongue, periodontal pocket (anaerobic surroundings), and carious lesions. Some bacteria act as commensals and provide essential health benefits to humans.^8^ Bacteria within a biofilm are resistant to antimicrobial agents than planktonic bacteria and escape the host immune responses.^9^

In light of this, the present study is aimed at developing a bioinspired antibiofilm polylactic acid (PLA) surface based on lotus and peepal leaves, validate its effect on attachment of opportunistic pathogens including *Staphylococcus aureus*, *Escherichia coli*, *Lactobacillus* sps., *Streptococcus mutans*, and *Pseudomonas aeruginosa*, and use a machine-learning algorithm to predict the role of surface roughness in biofilm formation.

*S. aureus* is a Gram-positive bacterium that causes several infections, including nosocomial and skin infections, endocarditis, and contamination in medical devices. Surface proteins that are covalently linked to peptidoglycans,^10^ including Bap (biofilm-associated protein), SasG (surface protein), CLLB (clumping factor), SdrC (serine aspartate repeat protein), FnBPA, IsdC (iron-regulated surface determinant protein C), Emp (extracellular matrix protein binding protein), Eap (extracellular adherence protein),^11^ and FnBPB (fibronectin/fibrinogen-binding proteins), promote biofilm formation in *S. aureus*. *E. coli* is a Gram-negative opportunistic most common bacterium found in biofilms that are associated with urinary tract infections. Ag43 (outer membrane protein that promotes aggregation and clump formation), AidA and TibA (glycosylated surface proteins),^12^ capsular polysaccharides, lipopolysaccharides, surface polysaccharides, and bundle-forming pili are some of the surface proteins that play a major role in the biofilm formation of *E. coli*. *Lactobacillus* sps. are Gram-positive bacteria that constitute the oral microbiota and are closely linked to the oral health of an individual.^13^ The genus *Lactobacillus* is the major source of probiotics. MabA protein plays a crucial role in initial adhesion and the S-layer protein forms a crystalline-lattice-like structure and enhances bacterial adhesion to surfaces. LuxS-derived autoinducer-2 (AI-2) acts as a chemical messenger between bacteria and initiates biofilm formation.^14^ *S. mutans* causes dental caries in the oral ecosystem, including *Lactobacillus* (causes dental lesions) and *Actinomyces* (affects root cementum), and acts as a stabilizer on dental caries. Acquired follicle formation followed by the adhesion of salivary glycoproteins on the tooth surface, electrostatic interactions, hydrophobic interactions, calcium bridges, chemical forces, and physical attachments^15^ plays an important role in the initial stages of biofilm formation, whereas mature biofilms have porous layers and water channels that provide essential nutrients to the cells.^16^ Bacterial species use several competitive mechanisms such as quantum sensing (QS), competence-stimulating peptide (CSP), hydrogen peroxide excretion, and bacteriocins to compete with other bacteria for survival, nutrients, and binding sites. Some proteins, including glucan-binding proteins, collagen-binding proteins, fibronectin-binding proteins, and glucosyl transferases, are involved in the formation of dental plaques on tooth surfaces, act as scaffolds for biofilm formation, and an escape mechanism from phagocytosis in *S. mutans*. *P. aeruginosa* is a well-known model to study biofilm formation that competes, dominates, and survives in the cystic fibrosis of the lung (polymicrobial environment). LecA and LecB (lectin proteins), CdrA^9^ fibrillar adhesion protein (bacterial aggregation formation), OprF (supports bacterial localization), three types of EPS, and alginate (matrix scaffold) are the biofilm-forming proteins associated with *S. mutans*. Several neural network models including multilayer perceptron (MLP) were tested to understand the complex nonlinear relationship between the surface charge of the polymers/bacteria and biofilm formation.

In light of this, we selected superhydrophobic lotus leaves that resist water and microbial adhesion, hydrophilic peepal leaves along with high-density polyethylene (HDPE), and polylactic acid. Bioinspired polylactic acid sheets mimicking lotus and peepal leaf surfaces were fabricated and tested for bacterial attachment. Bacterial adhesion was correlated with PLA hydrophobicity and surface roughness. Increased surface roughness is attributed to greater hydrophilicity identified by lower contact angle and higher biofilm formation, contrary to the hydrophobic surfaces. Applying machine-learning models offers mechanistic insights into bacteria–material interactions, and it aids in designing smart, surface-engineered antibacterial or bactericidal biomaterials for healthcare applications.

## 2 Materials and Methods

### 2.1 Synthesis of Lotus and Peepal Inspired Polylactic Acid Surfaces

To mimic lotus and peepal leaf surfaces, fresh lotus leaves were obtained from a lily pond at Thiruporur, Chennai, India, and peepal tree leaves were obtained from Centre for Biotechnology, Anna University, Chennai. Leaves were washed with sterile water and placed on sterile Petri dishes. 2% PLA (50 mL) was poured on the surface of the leaves and dried at room temperature overnight. After 12 h, the PLA sheet was peeled out from the lotus and peepal leaf surfaces and stored at room temperature. Sheets were named as PLA-LL (polylactic acid-lotus leaf), PLA-PL (polylactic acid-peepal leaf), HDPE (high-density polyethylene), and PLA (polylactic acid), and the latter two were used as controls to compare biofilm formation. The thickness of the sheet was measured using Vernier Callipers, and an average was taken.

### 2.2 Pretreatment of Bioinspired PLA Materials

PLA-LL, PLA-PL, plain HDPE, and PLA sheets were cut into 1 × 1 5 cm^2^ dimensions, treated with 70% isopropyl alcohol (IPA) for 15 min, washed twice with phosphate buffer saline (PBS), and kept under UV for 15 min for sterilization. Sheets were then transferred to a 24-well plate for the biofilm experiment.

### 2.3 Estimation of Bacterial Biofilm Formation—*S. aureus*, *E. coli*, *Lactobacillus* sps., *S. mutans*, and *P. aeruginosa*—Using Crystal Violet Assay

Crystal violet staining was performed to quantify the bacterial biomass formed on PLA-LL, PLA-PL, plain HDPE, and PLA surfaces. In a sterile 24-well plate, 1 × 1.5 cm^2^ of bioinspired sheets were placed and cultured with (1 × 10^–4^) of *S. aureus*, *E. coli*, *Lactobacillus* sps., *S. mutans*, and *P. aeruginosa* for 72 h (Figure S1). Later, the culture filtrate was removed carefully from the 24-well plate without damaging the bioinspired sheets. The sheets were washed three times with PBS gently, and the biofilm formed was stained with 0.1% crystal violet dye (1 mL) and left undisturbed for 4–5 min, followed by PBS wash. The biofilm-bound crystal violet dye was solubilized using 30% acetic acid (200 μL) (Figure S2), and the optical density was measured using a microplate reader at 595 nm (The Spark multimode microplate reader).^17^

### 2.4 Determination of Bacterial Hydrophobicity: Bath Assay

Overnight-grown culture of *S. aureus*, *E. coli*, *Lactobacillus* sps., *S. mutans*, and *P. aeruginosa* (2 mL or 0.8 OD) was centrifuged at 8000 rpm for 5 min. After centrifugation, the pellets were collected and solubilized using PBS (2 mL) and split into two volumes. One was transferred to a 96-well plate, and the optical density was measured using a microplate reader at 600 nm and labeled OD1, with PBS as blank. To the other volume, xylene (200 μL) was added to the remaining cells in the tubes, vortexed, and allowed for phase separation. Later the top phase was separated, and the optical density was measured and labeled OD_2_.^18^

The percentage of hydrophobicity was calculated from the following formula

$$\mathrm{percentage}\;\mathrm{of}\;\mathrm{hydrophobicity}=\left(\left(OD_{1}-OD_{2}\right)/OD_{1}\right)\times 100$$

### 2.5 Contact-Angle Measurements and Scanning Electron Microscopy

The wettability of the bioinspired sheets was determined using a Goniometer (Ossila Contact Angle Goniometer). The dried samples of polymeric sheets were cut into 1 × 1.5 cm^2^ dimension. A single drop of water was placed on the top surface of each polymeric sheet at room temperature, and the contact angle was measured with the help of a camera and software (Ossila Contact Angle v3.0.3.0-LM). The morphologies of PLA-LL and PLA-PL sheets were analyzed using a scanning electron microscope FEI Quanta FEG 200-High Resolution.

### 2.6 Surface Roughness Measurements

Surface roughness of the bioinspired sheets was measured using the Stylus profilometer-Mitutoyo SJ300 with an area size of 1 × 1.5 cm^2^ sheet. The measurement of surface roughness was repeated three times, and the Ra values were calculated based on an average of three individual measurements with each sheet.

### 2.7 Machine-Learning Tools

WEKA software^19^ was used to test machine-learning algorithms and data mining. Material surface roughness, surface hydrophobicity, contact-angle measurements, and organism’s hydrophobicity, together with the bacterial biomass formed, were used for predictive analysis. Several methods, including Random forest, J48, simple logistic regression, logistic regression, and multilayer perceptron (neural net) were used to classify the data^20^ using the respective toolbox in WEKA. Since contact angle and roughness are correlated, we considered organism hydrophobicity and roughness alone as the attributes (features) to predict the extent of biofilm formation.^19^ The efficiencies of various models tested were ascertained using different evaluation metrics including accuracy, root mean squared error (RMSE), Matthew’s correlation (MCC), and receiver operating characteristic (ROC) area. These terms are defined as follows: RMSE tells about the difference between the predicted and actual values; MCC is a measure of the quality of binary (two-class) classification (closer to 1.0 means highly correlated) and is similar to Pearson’s correlation coefficient; and ROC curve is a graphical representation of the trade-off between the false-negative and false-positive rates for every possible cut off and it is also a trade-off between sensitivity and specificity.

Logistic regression^21^ identifies the probability of an occurrence in the form of a discrete or categorical value by means of fitting data to a logistic function. J48 is a decision-tree-based classifier and classifies based on decision trees or rules generated from them. It builds decision trees based on the training data in the same way as the ID3 algorithm by using the concept of information entropy. ID3, Iterative Dichotomiser 3, is an algorithm which iteratively dichotomizes (divides) features into two or more groups at each node by iteratively selecting the best attribute to split the data based on information gain (improvement of ID3 algorithm) based on simplified information entropy and coordination degree.^22^ Each node represents a test on an attribute, and each branch represents a possible outcome of the test. The leaf nodes of the tree represent the final classifications.^23^ Random forest creates a set of decision trees from a randomly selected subset of the training set, and then it collects the votes from different decision trees to decide the final value, or it takes an average of all of the results from various decision trees (Random forests: from early developments to recent advancements^24^).

Multilayer perceptron is a feed-forward network trained using the back-propagation method.^25,26^ It consists of input, output, and several hidden layers (in between layers).

## 3 Results and Discussion

### 3.1 Hydrophobicity and Roughness of Bioinspired Polylactic Acid Sheets

The fabrication method is illustrated in Figure 1, and the flowchart shows the comprehensive methodology employed in our study to assess biofilm formation on biomimetic surfaces (Figure 2). Contact-angle (θ_R_) measurements revealed that PLA-LL θ_R_ is moderately hydrophilic (<68°), PLA-PL was highly hydrophilic (<44.6°), and HDPE and plain PLA had contact angles of <62 and 61°, respectively (Figure 3). Surface roughness varied from 0.88 for PLA-LL, and 2.43, 0.44, and 0.46 for PLA-PL, plain HDPE, and PLA, respectively.

**Figure 1:**
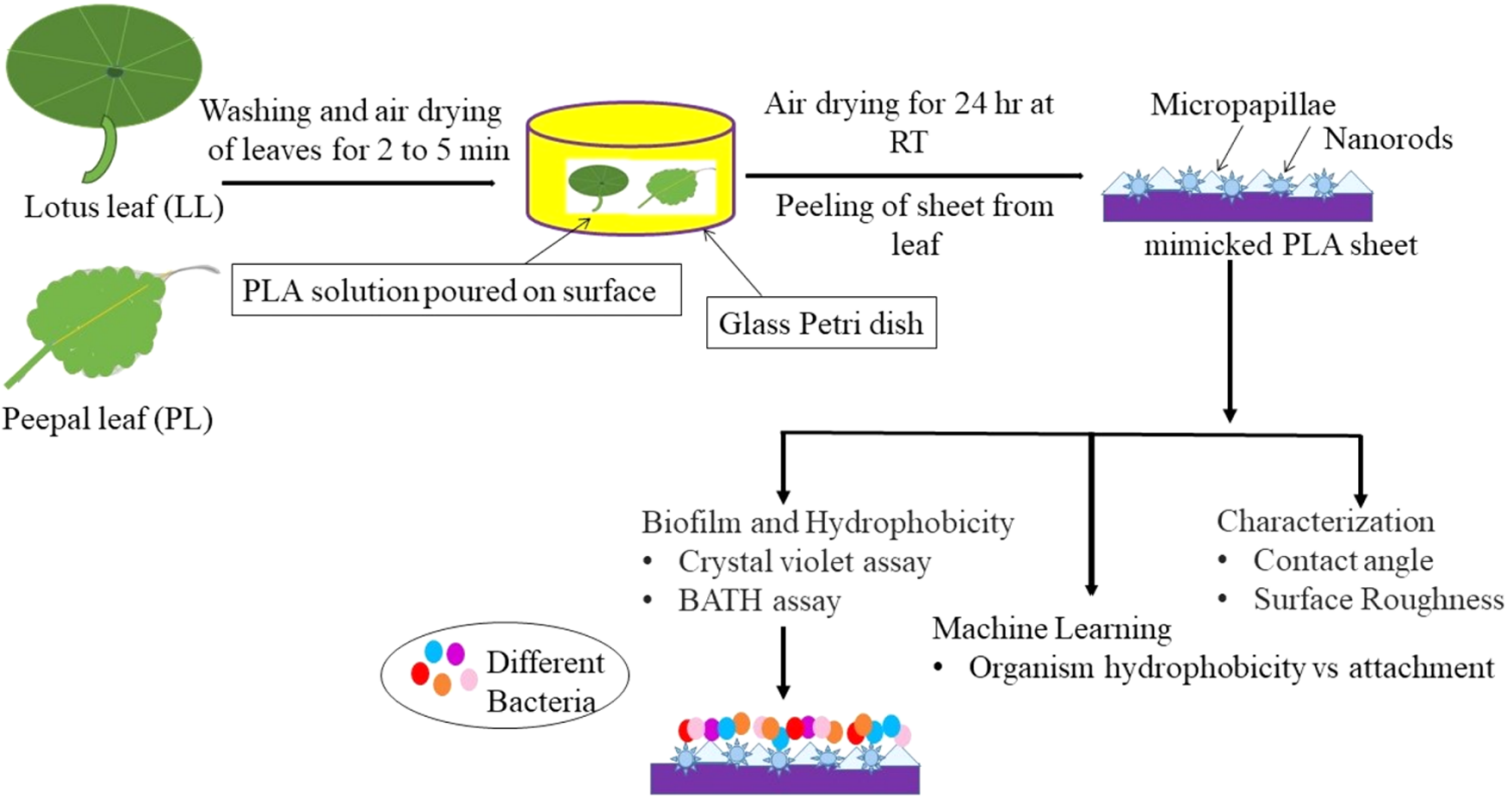
Schematic of the fabrication method to obtain the LL and PL surfaces.

**Figure 2:**
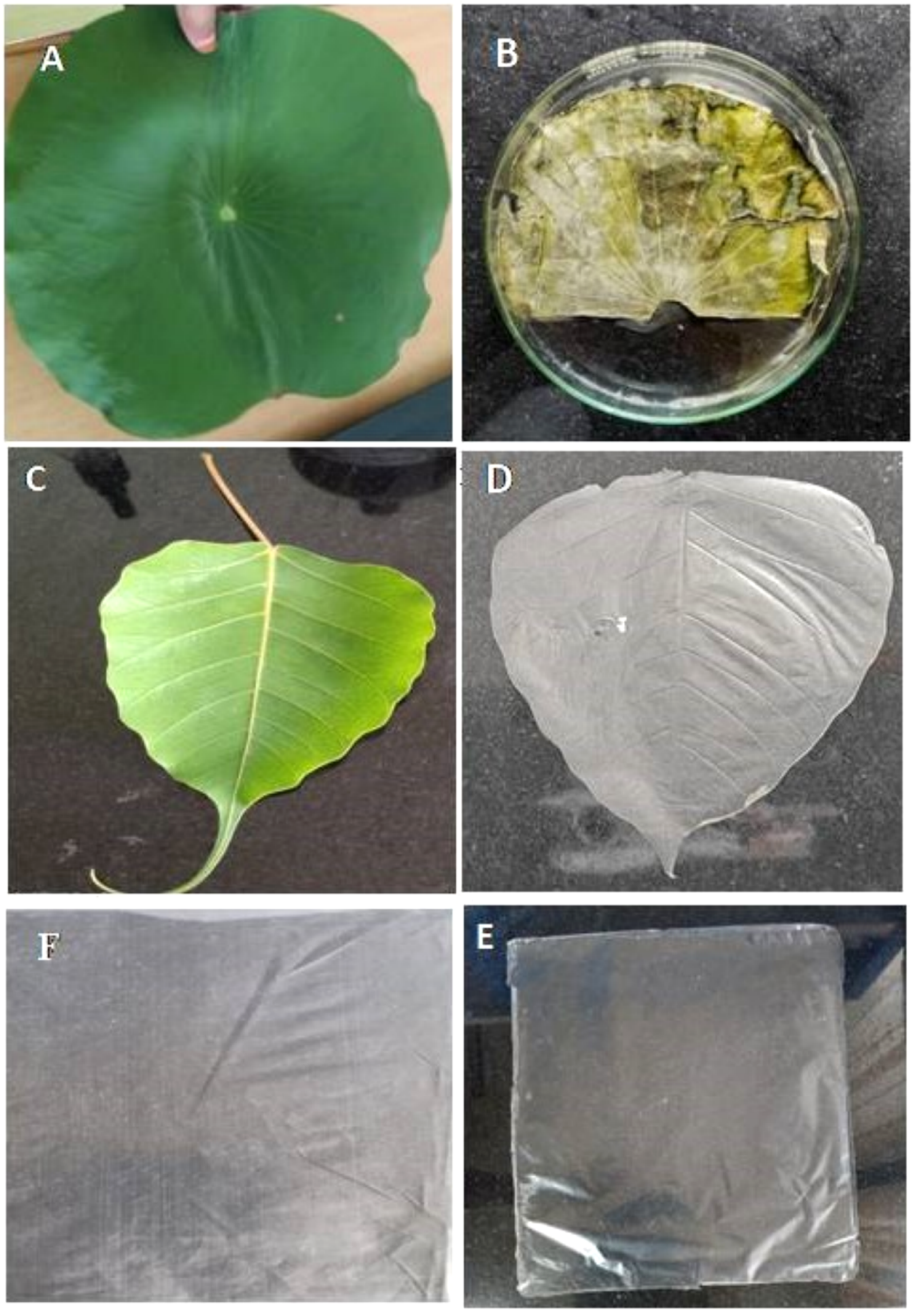
Photographic images of fresh lotus (A) and peepal (C) leaves and the respective bioinspired polylactic acid sheets (B, D) and control PLA (E) and HDPE (F).

**Figure 3:**
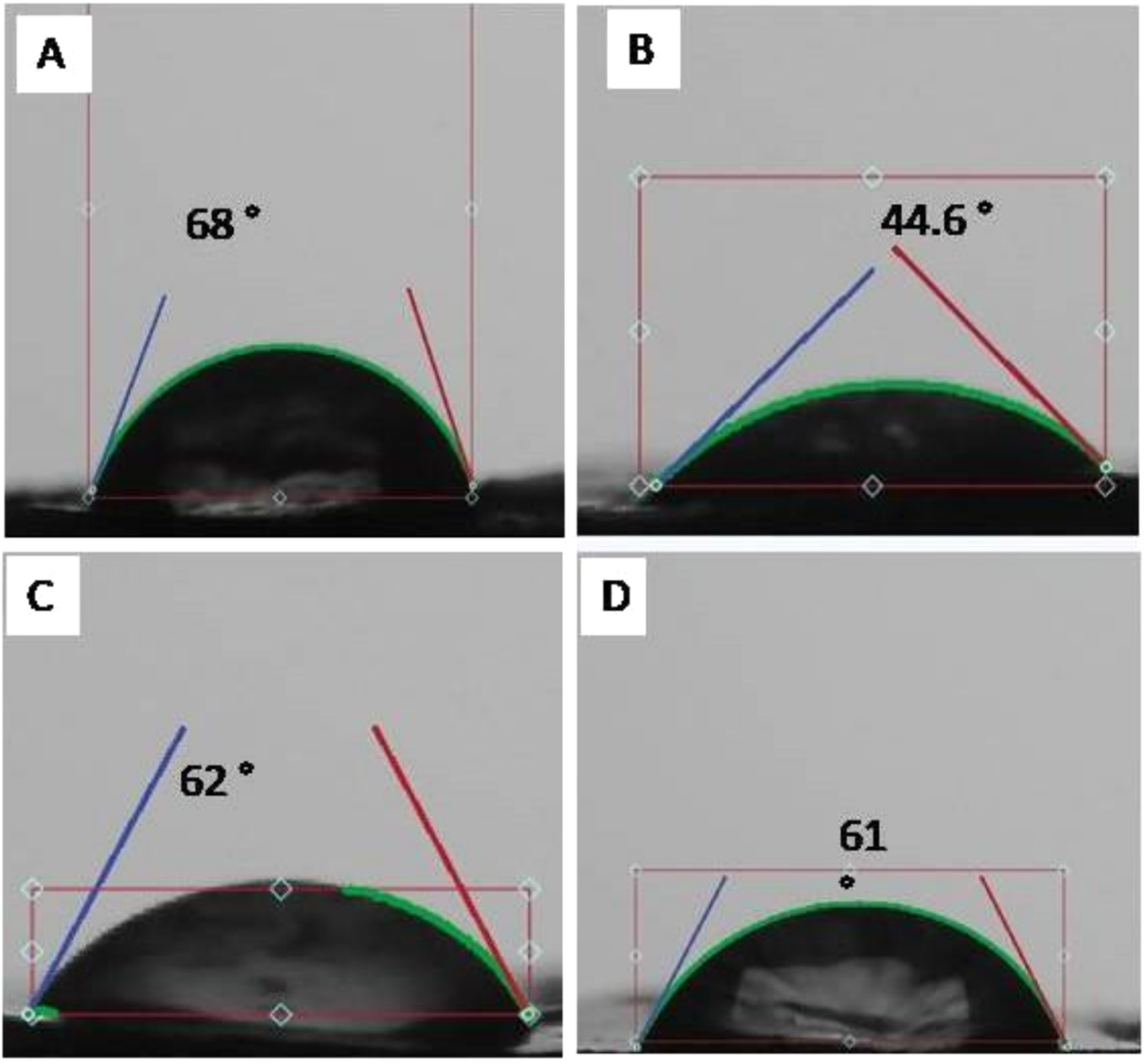
Contact-angle measurements of bioinspired polymeric sheets: (A) PLA-LL, (B) PLA-PL, (C) plain HDPE, and (D) PLA. PLA-LL θ_R_ is <68°, indicating moderate hydrophilicity. The PLA-PL surface showed a lower contact angle of <44.6°, suggesting lower hydrophilicity (higher hydrophilicity). HDPE and plain PLA have contact angles of <62 and 61°, respectively,, reflecting relatively higher hydrophobic characteristics.

These variations in surface topography are significant since it is known that increased surface roughness enhances bacterial adhesion by providing a large surface area, providing protective niches for microbial colonization, and by facilitating bacterial attachment, even on hydrophobic materials. Xu and Siedlecki^27^ demonstrated that increased surface roughness significantly influenced bacterial adhesion, with rougher surfaces promoting greater colonization by *Staphylococcus epidermidis*. Similarly, increased roughness on the hydrophilic surface promotes bacterial adhesion and biofilm formation.^28^

### 3.2 Relationship between Surface Roughness and Contact Angle

Strong correlation was observed between surface roughness, wettability, and biofilm formation. An increase in surface roughness leads to a decrease in the contact angle, making the surface more hydrophilic. Hydrophilic surfaces support biofilm formation, whereas hydrophobic surfaces reduce bacterial attachment (Table 1). Percentage hydrophobicity of the tested bacterial strains was measured by a Bath assay (Figure 4).

**Figure 4:**
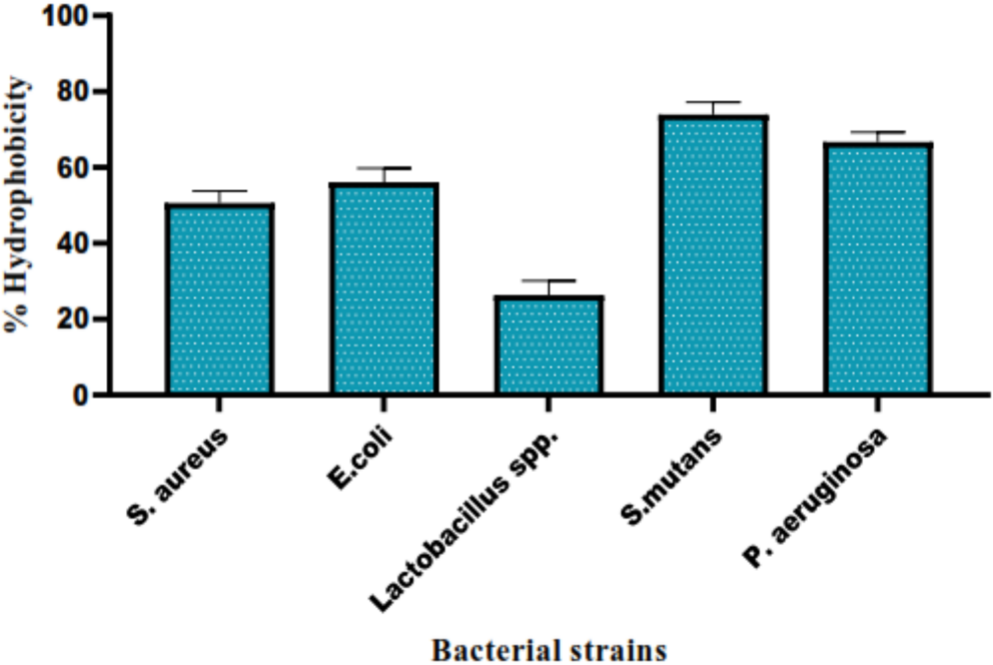
Percentage of hydrophobicity of different bacterial strains.

**Table 1:** Relationship between the Roughness of Polymers and Contact Angle

sl. no.	samples	contact angle	average surface roughness (Ra)	average biofilm (absorbance 595 nm)
1.	LL	68/0	0.88	0.070
2.	PL	44.6	2.43	0.097
3.	HDPE	62/0	0.44	0.046
4.	PLA	61/0	0.46	0.055

The thickness of the polymeric sheets was in the range of 20–50 mm (Figure S3). Electron microscopic images of the PLA-LL sheet revealed a hierarchical structure consisting of papillae, wax clusters, and wax tubules with a radius of 1.48–5.6 μm (Figure 5A,C) and a PLA-PL sheet with a radius of 5.52–8.27 μm (Figure 5B,D).

**Figure 5:**
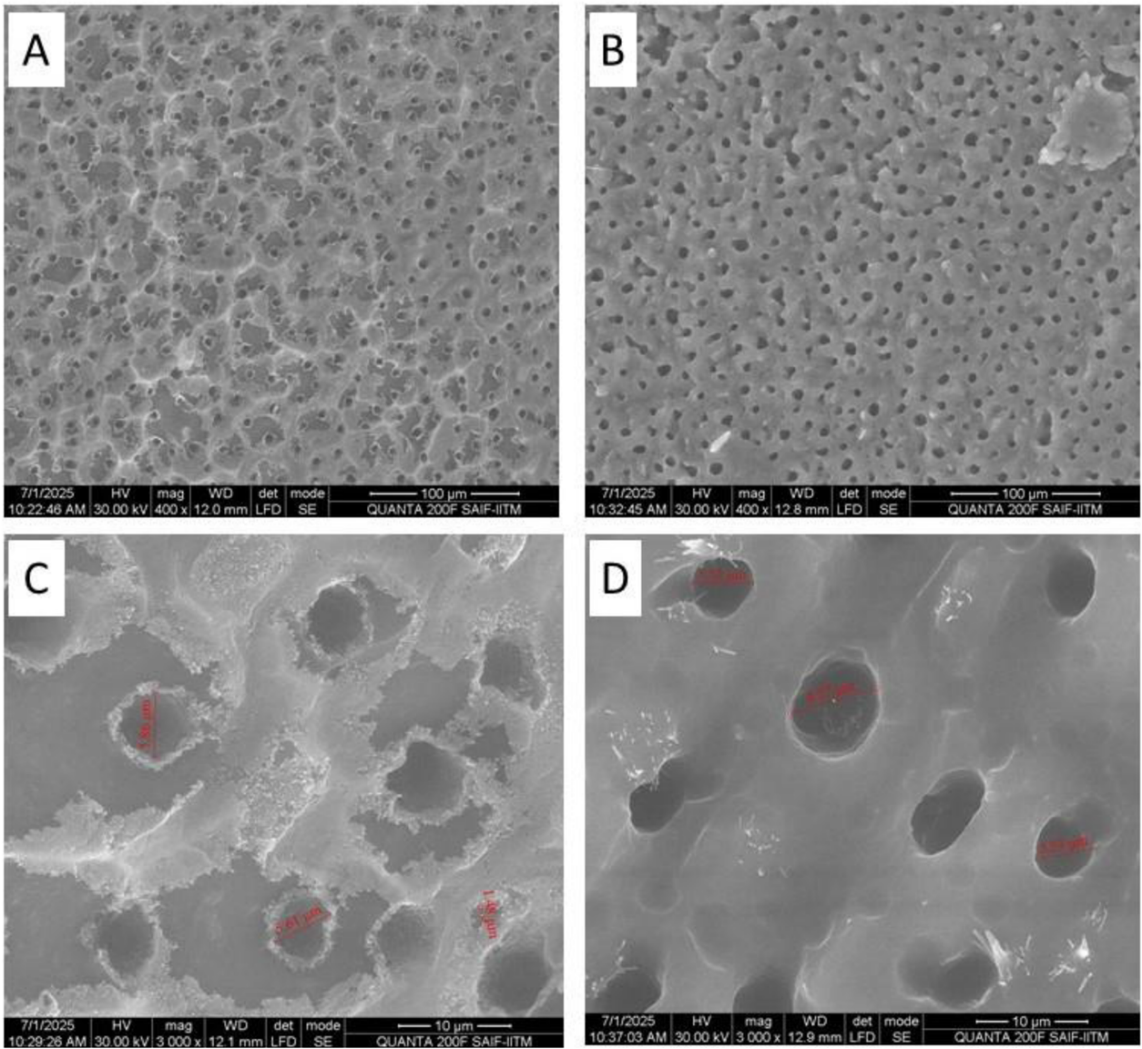
SEM image of lotus leaf mimicked PLA sheets. (A, C) PLA-LL sheet showing a hierarchical surface structure consisting of papillae, wax clusters, and wax tubules with a radius of 1.48–5.6 μm. (B, D) PLA-PL sheet with surface mimicked structures and a radius of 5.52–8.27 μm.

A negative correlation was observed between the contact angle and surface roughness (Ra) (*r* = −0.86), indicating that rougher surfaces are more hydrophilic. Similarly, there was a negative correlation between the contact angle and the average biofilm (*r* = −0.74). Hydrophobic surfaces tend to resist initial bacterial adhesion due to lower surface energy and reduced interaction with the aqueous environment, which supports previous findings on the antifouling properties of hydrophobic materials.^32,33^ For example, LF-inspired surfaces have been widely studied for their self-cleaning and antibiofouling characteristics due to their high water repellence and low bacterial adhesion.^34^ There was a positive correlation between surface roughness and biofilm (*r* = −0.97). This is in line with previous studies, indicating that surface irregularities provide more surface area and shelter for bacteria, thus facilitating initial adhesion and subsequent biofilm growth.^30,31^ Rough micro- and nanoscale features can protect bacteria from shear forces and improve mechanical anchorage, contributing to the persistence of biofilms.

Our results revealed that rough surfaces promote greater biofilm accumulation, whereas smoother surfaces inhibit it, suggesting a strong interplay among surface roughness, wettability (contact angle), and biofilm formation on polymeric materials. The negative correlation between roughness (Ra) and contact angle (*r* = −0.86) suggests that increasing the surface roughness of polylactic acid sheets enhances their hydrophilicity. This is in agreement with Wenzel’s model, describing how surface roughness amplifies the intrinsic wettability of a material, making hydrophilic surfaces more hydrophilic and hydrophobic ones more hydrophobic.^29^ A significant positive correlation was observed between surface roughness and biofilm formation (*r* = 0.97), indicating that rougher surfaces favor microbial attachment and biofilm development. Rough micro- and nanoscale features can protect bacteria from shear forces and improve mechanical anchorage, contributing to the persistence of biofilms. Conversely, surfaces with higher contact angles (more hydrophobic) were associated with decreased biofilm accumulation (*r* = −0.74). These results suggest that tailoring the surface properties of biomaterials, such as controlling the roughness and hydrophobicity, can be a viable strategy for managing biofilm formation. Applications in biomedical implants, food packaging, and water treatment systems can greatly benefit from materials engineered to reduce the level of bacterial colonization.

### 3.3 Organism Hydrophobicity vs Attachment on Various Surfaces

Biofilm formation was significantly influenced by both the physicochemical properties of the polymer surfaces (*p* < 0.005) and surface hydrophobicity of the bacteria (*p* < 0.0001) (Table 2). Bacterial adhesion is largely determined by the proteins on their surfaces. A weak correlation (*r* = 0.55) was observed between the percentage hydrophobicity of the organisms and the average biofilm formed on the surfaces tested (Figure 6), suggesting that bacteria with high surface hydrophobicity tend to form more substantial biofilms, particularly on surfaces that complement their hydrophobic nature. This is in line with previous studies indicating that bacterial adhesion and subsequent biofilm formation are influenced by the interaction between the hydrophobic properties of both the bacterial cell surface and the substratum.^35^ *S. mutans* showed maximum adhesion, while *E. coli* exhibited the least, which could be attributed to differences in their cell surface properties because *E. coli* is more hydrophilic than *S. mutans*, exhibiting enhanced attachment.^36^ These observations underscore the importance of considering both bacterial surface characteristics and material properties when designing surfaces intended to minimize biofilm formation.

**Figure 6:**
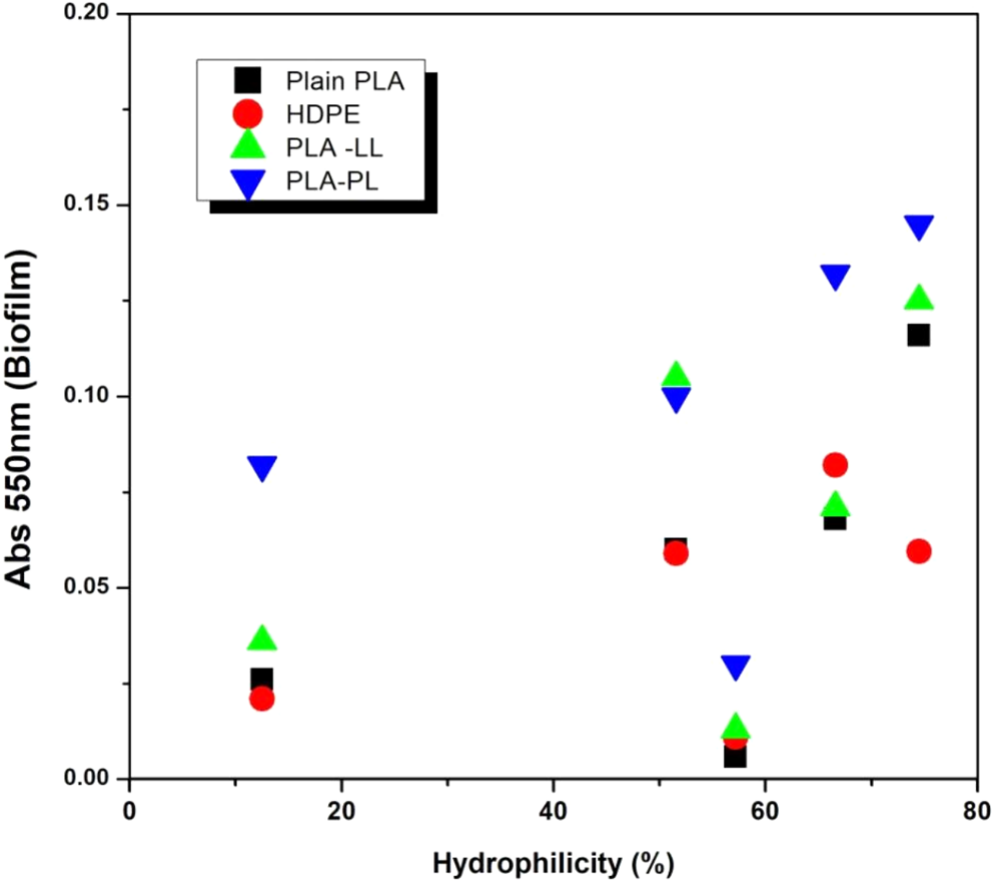
Organism hydrophobicity (%) vs attachment on surfaces like PLA-LL, PLA-PL, HDPE, and plain PLA in a 24-well microtiter plate inoculated with grown cultures (1 × 10^–4^) and incubated for 73 h. Then, the biofilm was stained with 1 mL of 0.1% crystal violet dye and the absorbance was measured at 595 nm by The Spark multimode microplate reader.

**Table 2:** Organism Hydrophobicity vs Attachment on Various Surfaces

attachment to various surfaces						
organism tested	% organism hydrophobicity	PLA-plain	PLA-lotus	PLA-peepal	HDPE	average attachment (absorbance 595 nm)
*P. aeruginosa*	66.58	0.07	0.07	0.13	0.08	0.09
*S. aureus*	51.58	0.06	0.11	0.10	0.06	0.08
*E. coli*	57.18	0.01	0.01	0.03	0.01	0.01
*Lactobacillus*	12.50	0.03	0.04	0.08	0.02	0.04
*S. mutans*	74.49	0.12	0.13	0.15	0.06	0.11
	average attachment	0.06	0.07	0.10	0.05	
	correlation with the organism’s hydrophobicity	0.60	0.51	0.42	0.57	0.55

### 3.4 Machine-Learning-Based Prediction

Several classifier models were used to predict biofilm formation based on the organism’s surface hydrophobicity and the surface roughness of the material. The evaluation metrics for each classifier tested are summarized in Table 3. The multilayer perceptron classifier, configured with two hidden layers containing two neurons each, demonstrated consistent performance across both training and cross-validation data sets, achieving an accuracy of approximately 85% (Figure 7A), indicating a balanced model with good generalization capabilities. In contrast, the Random Forest classifier achieved the highest accuracy (100%) on the training set but showed a significant reduction in accuracy to 70% during cross-validation. Other classifiers, including J48 and simple logistic, exhibited moderate performance, with accuracies ranging from 65 to 80% across different test options (Figure 7B). Each node in one layer connects with a certain weight to every node in the following layer. Learning occurs in the perceptron by changing connection weights after each piece of data is processed based on the amount of error in the output compared to the expected result.

**Figure 7:**
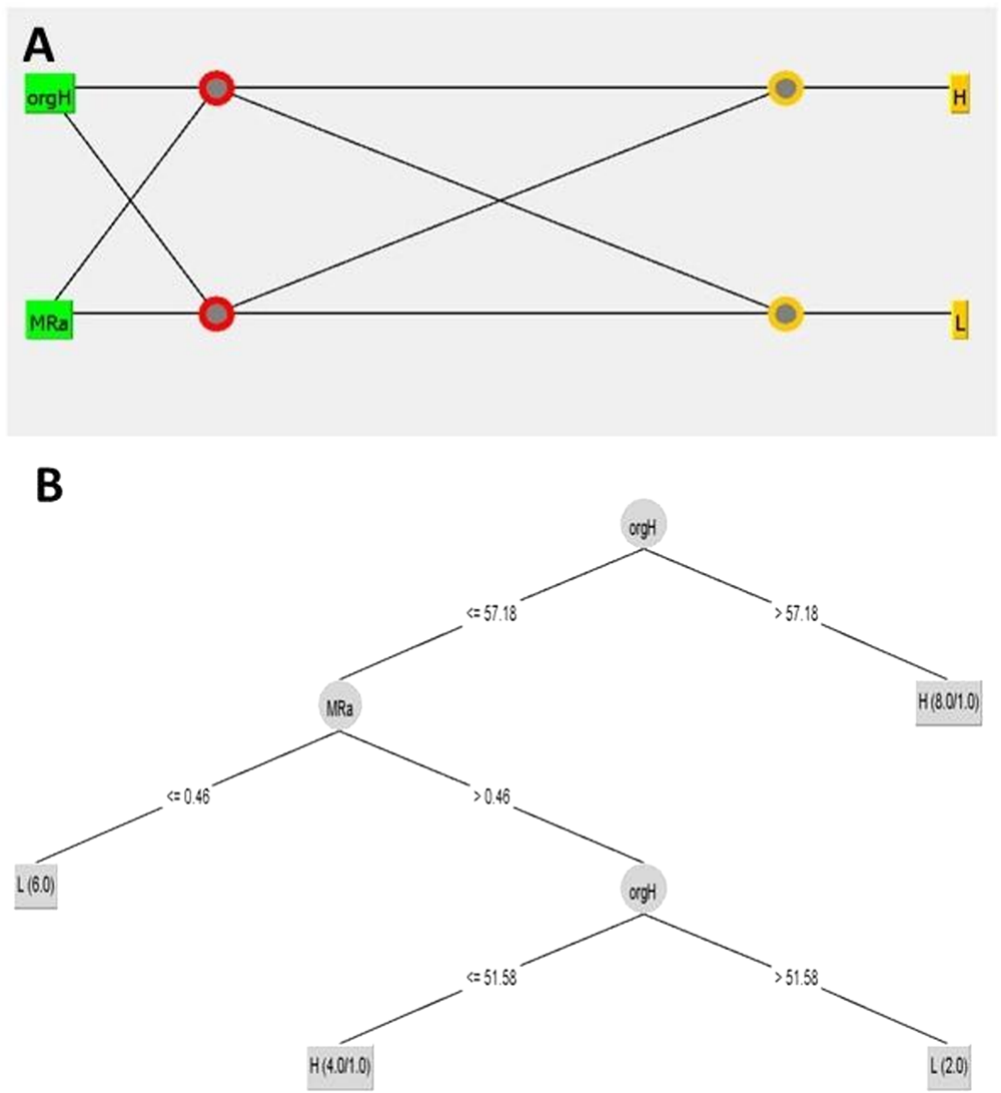
(A) Multilayer perceptron (neural network model). Organism surface hydrophobicity and material surface roughness are the two inputs, and the extent of biofilm is the output. (B) Classifier tree based on the J48 method (orgH = hydrophobicity of the organism surface, MRa = material roughness, H = high biofilm, L = low biofilm). If organism surface hydrophobicity is >57%, we observe high biofilm formation. If this value is less than 57%, then low roughness of the polymer (roughness < 0.46) leads to the formation of low biofilm. So, very smooth surfaces lead to low attachment (as reported in the literature). We have no control over the organism’s surface hydrophobicity since the type of organism present in the environment depends on the location (in the host) where the biomaterial is placed in the body. If the environment may have organisms with high surface hydrophobicity, then biomimetic surfaces alone cannot reduce the biofilm formation, but we may require antibacterial or antibiotic coatings to kill them.

**Table 3:** Results of the ML Using Various Classifiers with Corresponding Statistics

classifier	test option	accuracy	RMSE^c^	MCC^d^	ROC area^e^
random forest	training set^a^	100	0.158	1.0	1.0
	cross-validation (5 folds)^b^	70	0.428	0.4	0.85
J48	training set	90	0.285	0.816	0.92
	cross-validation (5 folds)	65	0.57	0.3	0.6
simple logistic	training set	75	0.41	0.50	0.83
	cross-validation (5 folds)	80	0.47	0.6	0.78
logistic regression	training set	65	0.41	0.3	0.84
	cross-validation (5 folds)	70	0.46	0.4	0.76
multilayer perceptron (neural net)	training set	85	0.35	0.73	0.86
	cross-validation (5 folds)	85	0.41	0.73	0.76

a The entire training set data was used to train the model: No validation.

b 90% of the data was used for training and the remaining was used as a test set. This was done five times and averaged out.

c Root mean squared error.

d Mathew’s correlation coefficient varies from −1 to 1. It is used in machine learning as a measure of the quality of binary and multiclass classifications. It produces a high score only when the prediction obtained good results in all four confusion matrix categories (false-positive, false-negative, true-positive, true-negative).

e A receiver operating characteristic curve, a graphical plot that illustrates the performance of a binary classifier model at varying threshold values. The ROC curve is the plot of the true-positive rate against the false-positive rate at each threshold setting.

## 4 Conclusions

Bioinspired polylactic acid sheets mimicking lotus and peepal leaf surfaces were fabricated and tested for bacterial attachment. Bacteria with higher surface hydrophobicity tend to form more substantial biofilms, particularly on surfaces that complement their hydrophobic nature. A negative correlation was observed between contact angle and surface roughness (Ra) (*r* = −0.86), indicating that rougher surfaces are more hydrophilic. Similarly, there was a negative correlation between the contact angle and average biofilm. Machine-learning algorithms can handle complex, multidimensional data sets, uncovering patterns and relationships that may not be obvious through traditional statistical methods. ML and deep learning algorithms have been used to analyze biofilm images, facilitating the quantification of biofilm characteristics and enhancing our understanding of biofilm dynamics. In the present study, our focus was to predict the role of surface roughness and hydrophobicity. A strong correlation was observed between the surface roughness, wettability of bioinspired polymers, and biofilm formation.

Incorporating multiple features enhances the predictive power of ML models. The multilayer perceptron (MLP) classifier was accurate in predicting biofilm formation based on surface properties. These models could aid in the design of biomaterials and implants with tailored surface characteristics to minimize biofilm-related complications. Future research should focus on expanding the range of input features, increasing the data set, and validating the models across diverse biological and environmental contexts to enhance their applicability in real-world scenarios.

## Supplementary Material



## Notes

Cultured sheets in 24-well plates, CV assay (24-well), and thickness of the film (PDF)

## ⊥

Indian Institute of Science, Bangalore 560012, India

## ○

Indian Institute of Technology, Jodhpur 342030, India

## #

National Institute of Pharmaceutical Education and Research, Mohali, Punjab 160062, India
