# Supplementary material for: Biofilm-Forming Ability of Infectious Organisms on Biomimetic Surfaces—An In Vitro and Machine-Learning Analysis
Source: ACS Omega. 2025 Aug 25;10(35):39946–54. doi: 10.1021/acsomega.5c04335 (PMC12423850; doi:10.1021/acsomega.5c04335)

Supporting Information ACS Omega

Biofilm Forming Ability of Infectious Organisms on Biomimetic Surfaces – An *In Vitro* and Machine Learning Analysis

Geetha Venkatachalam[a], Nandakumar Venkatesan[b], Shloak Vatsal[c], Indira Chavan[c], Arnab Bakshi[c], Mukesh Doble[d]*

[a]Ecogreen Innovations Pvt Ltd, Nirmaan, The Pre-incubator, Sudha Shankar Innovation Hub, IIT Madras, Chennai,India.

[b]Sri Ramachandra Faculty of Engineering and Technology, Sri Ramachandra Institute of Higher Education and Research, Chennai, India.

[c]Theevanam Additives and Nutraceuts Pvt. Ltd, IITM Research Park, Chennai,India.

[d]Department of Cariology, Saveetha Dental College, SIMATS, Chennai,

India. E-mail: mukeshdoble.sdc@saveetha.com

***Corresponding Author**

Prof. Mukesh Doble

Department of Cariology, Saveetha Dental College, SIMATS, 600077, Chennai,

Tamilnadu, India.

E-mail: mukeshdoble.sdc@saveetha.com

Figure S1, Sheets with culture in 24-wellplate

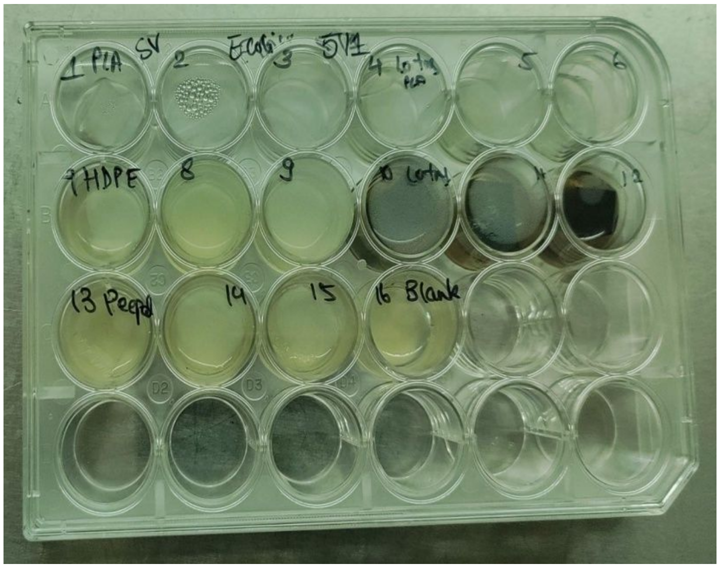

Figure S2, Crystal Violet assay in 24 well plate

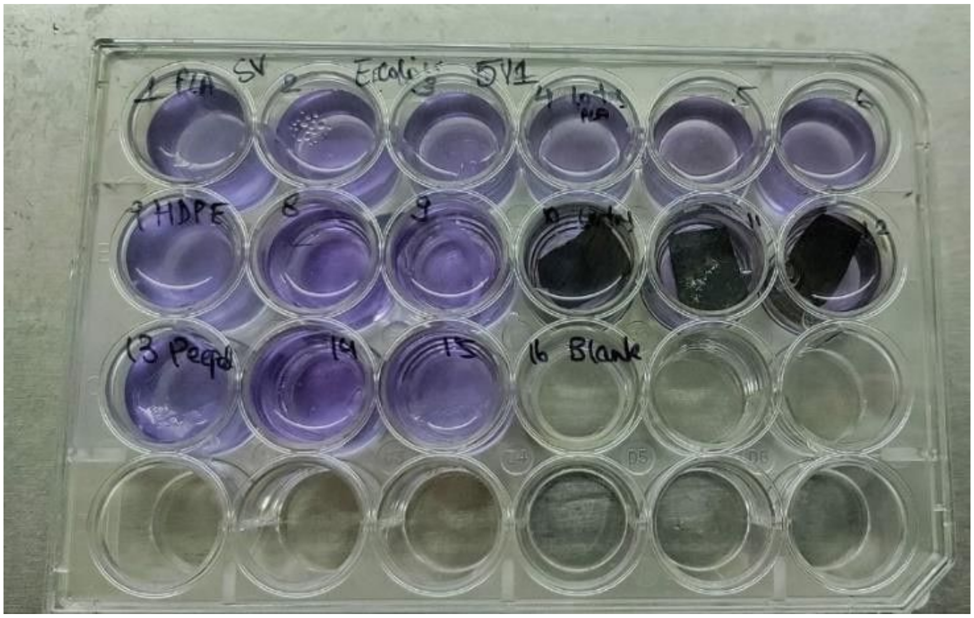

Figure S3, Thickness of the polymeric sheet.

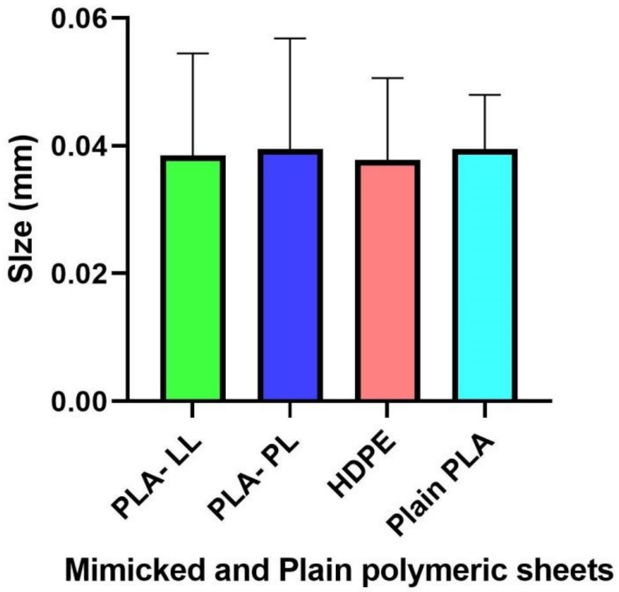

Supplement: Supplementary file 1 [file ao5c04335_si_001.pdf]
